# Anti-cancer strategies targeting the autotaxin-lysophosphatidic acid receptor axis: is there a path forward?

**DOI:** 10.1007/s10555-021-09955-5

**Published:** 2021-01-17

**Authors:** Gábor Tigyi, Mélanie A. Dacheux, Kuan-Hung Lin, Junming Yue, Derek Norman, Zoltán Benyó, Sue Chin Lee

**Affiliations:** 1 Department of Physiology, University of Tennessee Health Science Center Memphis, 3 N. Dunlap Street, Memphis, TN 38163 USA; 2 Department of Pathology, University of Tennessee Health Science Center Memphis, 3 N. Dunlap Street, Memphis, TN 38163 USA; 3 Institute of Translational Medicine, Semmelweis University, POB 2, Budapest, H-1428 Hungary

*“LPA-based cancer therapeutics: is there a path forward?”*

The lipid mediator lysophosphatidic acid (LPA) was first identified more than thirty years ago as an agent regulating multiple physiological responses including platelet aggregation, blood pressure, contraction of smooth muscle cells, cell shape, and neurite outgrowth. In the last two decades, discoveries of autotaxin (ATX)—the major LPA-producing lysophospholipase D enzyme—and the family of six LPA G protein coupled receptors (GPCR, namely LPAR1–6) with omnipresent roles in malignancies from cell transformation, proliferation, invasion, and metastasis to survival against genotoxic or metabolic stress, rapidly propelled LPA to the center stage of cancer research. These observations collectively suggest that cancer cells have hijacked LPA to enhance tumor progression, metastasis, and therapy resistance. In spite of this, one major question remains: why are there no drug candidates targeting the ATX-LPAR axis approved for the treatment of cancer? To address this question, we need to first understand the two major challenges in cancer treatment—therapy resistance and the impact of the tumor microenvironment (TME) on cancer progression and therapeutic response.

## LPA: an omnipotent oncogenic and prometastatic lipid mediator

Researchers have, in large part, attributed therapy resistance to a rare subpopulation of tumor cells termed cancer stem cells (CSC), which exhibit unique properties including self-renewal, multi-lineage differentiation, and upregulation of drug efflux transporters. These characteristics make CSC inherently resistant to conventional chemotherapeutic agents. Moreover, their slower proliferative capacity is an added advantage since chemo- and radiation therapy are more effective against rapidly dividing cancer cells. Thus, increasing efforts are being directed at identifying and developing new therapeutics that selectively target CSC. Recently, it was demonstrated that LPA stimulates the expression of CSC-associated genes, including *OCT4*, *SOX2, ALDH1*, and drug transporters. ATX is also highly expressed in tumor sphere-forming CSC and primary ovarian CSC isolated from patients [1]. Emerging *in vitro* data demonstrate that inhibiting ATX, LPAR1, or LPAR2 reduces the acquisition of CSC-like properties in ovarian and breast carcinomas [1, 2]. CSC-targeted delivery of compounds inhibiting ATX or LPAR could be a viable new treatment strategy for therapy-resistant cancers.

Besides targeting CSC, the TME provides many opportunities for new the development of therapeutics. We have learned that the dynamic interaction between tumor cells with blood and lymphatic vessel networks, stromal elements of the extracellular matrix, fibroblasts, adipocytes, and immune cells ultimately dictates cancer progression and influences therapeutic outcomes. In this regard, the ATX-LPAR axis has recently been recognized as a central regulator of cells in the TME and metastatic niche. Breast cancer cells have been shown to reprogram adjacent adipocytes to increase production of ATX [3]. In blood, ATX is taken up and stored in the α-granules of platelets and is secreted upon tumor cell–induced platelet aggregation. The resultant localized generation of LPA promotes colonization of breast cancer cells to the bone [4] that is blocked by ATX inhibition. Studies showed that pancreatic ductal adenocarcinoma (PDAC) cells transform tissue-resident pancreatic stellate cells into activated cancer-associated fibroblasts (CAF) with an altered lipidome, in which the level of the LPA precursor lysophosphatidylcholine become significantly elevated. As PDAC cells overexpress ATX and convert CAF-derived LPC into LPA, which further drives and amplifies PDAC progression [5].

Collectively, these findings place the ATX-LPAR axis in a unique position where an opportunity exists to target both cancer and the TME cells. The big question is how?

## ATX or LPAR5/6 inhibitors as potential allies for immunotherapy

Cancer immunotherapy is one example of tumor treatment targeting the TME. The profound actions of LPA have been overshadowed by the therapeutic advances targeting the related lysophospholipid sphingosine-1-phosphate and its GPCR. In the context of tumor immunity, the effects of LPA on macrophages/dendritic cells and T cells offer enormous potential for therapeutics. Recent reports provided evidence that LPA inhibits the effectors of immunotherapy [6, 7]. Thus, the hypothesis of combining immunotherapy with ATX and/or LPAR inhibitors appears intriguing because the ATX-LPAR axis augments cancer cell progression and can directly attenuate tumor immunity by blocking effector CD8^+^ T cells, NK cells, and regulating tumor-associated macrophages (TAM). In particular, TAM, which constitute one of the most abundant immune cell populations in the TME, are typically associated with poor prognosis. Because LPA can convert monocytes into macrophages, it has been proposed [8] that in ovarian cancer, LPA could recruit monocytes and directly convert them into TAM within the TME *via* the PI3K/AKT/mTOR signaling pathway. As a vicious cycle, TAM-derived LPA in turn could promote metastasis and invasion of CSC. This hypothesis seems plausible and could point to the need of targeting the ATX-LPAR axis in the CSC. However, concrete proof of this mechanism remains to be provided.

It is now established that LPA exerts immunosuppressive effects in the TME. This finding was pioneered by the Torres group when they discovered that LPAR5 blocks the anti-tumor effector functions of CD8^+^ T cells [7]. Seeding and development of lung metastases in LPAR5 knockout mice were reduced by 85%, and adoptive transfer of CD8^+^ T cells isolated from *Lpar5*^−*/*−^ mice exerted enhanced killing of both EG7 lymphoma and B16F10 melanoma tumors compared to wild type CD8^+^ T cells. Conversely, stimulation of LPAR5 in human lymphocytes inhibited the allogeneic killing of SUM159.RFP human breast cancer cells *in vitro*. LPAR5 blocks the activation of the immunological synapse between the CD8^+^ T cell and its target cell, leading to the concept that LPAR5 appears to function as an immunological checkpoint controller. This hypothesis adds emphasis to development of LPAR5-specific inhibitors. Single-cell RNA sequence analysis of 32 clinical melanoma patient samples showed an inverse correlation of ATX expression in tumor cells with the number of tumor-infiltrating CD8^+^ T lymphocytes (TIL). Recently, it has been proposed that LPAR6 expressed by CD8^+^ T cells mediates the accumulation of human CD8^+^ TIL melanomas [6].

It is becoming increasingly convincing that combining ATX-LPAR inhibitors with current cancer therapeutics holds promise. However, a better understanding of the detailed function of each LPAR in different cancer and cell types is required. For example, a recent *in vivo* study that investigated the role of LPA in combination with anti-programmed cell death-1 (PD1) therapy reported that LPAR4 mediates the formation of an organized vascular network within brain tumors. This network led to an increase in TIL and enhanced the efficacy of anti-PD1 therapy [9]. In this particular context, inhibition of LPAR4 would have been deleterious. Therefore, the need to develop specific LPAR inhibitors is now compelling.

## Is there a path forward?

Finally, the looming question remains: what combination of therapies would be suitable to efficiently inhibit the “bad” components of the TME and LPA actions in carcinoma cells while simultaneously improving the efficacy of antitumor effectors to achieve a complete destruction of tumors? How will inhibiting the LPA-regulated components of the TME or blocking LPA production by ATX affect the other components as a result of continuous crosstalk between LPAR-regulated targets? The critical challenge lies in the lack of our understanding of the organ-specific tumor features and the individual antitumor immune response at the core of therapy. Another impediment is the lack of preclinical models that are representative of human cancer immunity. Furthermore, due to genetic instability of cancer, our current animal models are unable to mimic the complex changes taking place in human cancers.

There are two problems that the field must overcome: (1) The overlapping LPAR signaling pathways; and (2) The co-expression of multiple different LPAR in the same target regardless of whether it is a carcinoma or a TME cell. There are many types of cells within the TME in which LPAR actions have not been explored for therapeutic purposes due to a lack of knowledge of their ATX-LPAR phenotype and functions; how do they contribute to the progression of cancer? What are the underlying mechanisms of ATX upregulation and the reprogramming of the TME?

Nonetheless, it is clear that the ATX-LPAR axis plays a fundamental role in various aspects of cancer development. Perhaps it is the broad-range of LPA actions that is the barrier to devising action-targeted therapeutic interventions. In order to move forward, we need to develop new drug candidates that are specific to each individual LPAR, particularly the non-EDG type receptors. We also need to reach beyond pharmacological agents and develop cell-targeted delivery methods and interventions including siRNA-based silencing methods to key signaling and regulatory steps in the ATX-LPAR axis. Preventing the upregulation of LPAR and ATX by silencing regulators of their transcription in neoplastic cells and dysregulated cells of the TME might provide a yet unexplored avenue to therapy. A major hurdle is that LPA is an important mediator of multiple physiological responses, including development, wound healing, vascular reactivity, natural apoptosis, and reproductive function. Thus, blocking LPA generation globally can lead to unwanted side effects. Hence, devising LPA-based therapies all comes down to novel, LPAR-targeted approaches that increase the specificity of the therapeutic intervention without negating its physiological functions.

Having studied lysophospholipids for over three decades, these novel functions of LPA signaling and its interplay with multiple other signaling pathways continue to be a source of fascination, opening doors to new hypotheses and discoveries that will hopefully lead to many new clinical applications in cancer. There is no doubt that this is truly an exciting time to be in the field of lysophospholipid research.
